# Gray platelet syndrome as a multisystem disorder: immune dysregulation, autoimmunity, and metabolic abnormalities

**DOI:** 10.1007/s00277-026-07007-y

**Published:** 2026-04-14

**Authors:** Hussam Alzeerelhouseini, Leen Aljunaidi, Mays Alhouseini, Hani Saleh

**Affiliations:** 1 Faculty of Medicine, Al-Quds University, Main Campus, Abu Dis, P.O. Box 89, Jerusalem, Palestine; 2 College of Medicine and Health Sciences, Palestine Polytechnic University, Hebron, Palestine; 3 Department of Pediatric Hematology/oncology, Augusta Victoria Hospital, Jerusalem, Palestine

**Keywords:** Gray platelet syndrome, NBEAL2, Thrombocytopenia, Autoimmune

## Abstract

Gray platelet syndrome (GPS) is a rare inherited platelet disorder caused by defective α-granule biogenesis. Emerging evidence suggests that GPS represents a multisystem disorder with immune and inflammatory manifestations. We report three siblings from a single family with genetically confirmed GPS who presented with lifelong thrombocytopenia and mild to severe bleeding symptoms. Notably, all affected individuals had persistently elevated serum vitamin B12 of unclear etiology. One sibling had concomitant type 1 diabetes mellitus and biopsy confirmed celiac disease. This familial case series highlights the phenotypic heterogeneity of GPS and supports its recognition as a multisystem disorder. The consistent finding of hypercobalaminemia represents a notable but poorly understood feature, while the coexistence of autoimmune disease suggests underlying immune dysregulation related to NBEAL2 deficiency. Gray platelet syndrome may involve metabolic and autoimmune manifestations beyond bleeding. Awareness of these associations is essential for comprehensive evaluation and long-term management of affected patients.

## Introduction

Gray platelet syndrome (GPS) is a rare inherited platelet disorder with a limited number of reported cases worldwide. It is characterized by macrothrombocytopenia and absence or marked reduction of platelet α-granules, resulting in enlarged pale gray platelets on peripheral blood smear [1, 2]. GPS is inherited in an autosomal recessive pattern and is caused by pathogenic variants in the NBEAL2 gene, which is essential for α-granule biogenesis in megakaryocytes and platelets [3, 4].

Emerging evidence suggests that GPS is not limited to a bleeding disorder but rather represents a multisystem disease. Reported associations include myelofibrosis, splenomegaly, immune dysfunction, recurrent infections, and autoimmune manifestations, suggesting underlying immune dysregulation [5–7]. Elevated serum vitamin B12 levels have been described in several hematologic and inflammatory conditions, including disorders associated with myeloproliferation and immune activation; however, their occurrence and clinical significance in GPS remain poorly understood [8–10].

In this article, we describe a familial case series of GPS with autoimmune and metabolic associations and review the literature to further expand the recognized clinical spectrum of the disorder. To our knowledge, this is the first reported case of GPS associated with both type 1 diabetes mellitus and celiac disease, further supporting the hypothesis that GPS may involve systemic immune dysregulation.

## Case presentation

**Fig. 1:**
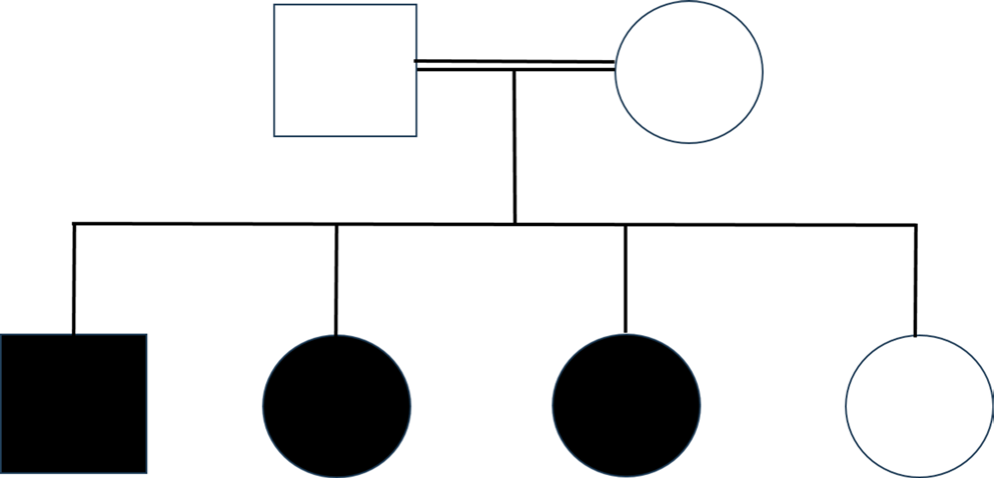
Pedigree of the reported family. Squares represent males and circles represent females. Filled symbols indicate affected individuals diagnosed with gray platelet syndrome

The patients were three affected siblings from a consanguineous family with a strong history of abnormal bleeding. The father and another sibling were clinically unaffected. The mother reported a history of easy bruising after minor trauma, while several paternal relatives including an uncle, grandmother, and three cousins had a history of recurrent bruising and prolonged epistaxis, suggesting an inherited bleeding disorder. The pedigree (Fig. 1) illustrates the segregation of Gray Platelet Syndrome within the family and highlights affected versus unaffected members.

The clinical features, hematologic findings, serum vitamin B12 levels, and associated autoimmune conditions of the three affected siblings are summarized in Table 1.

**Table 1:** Summarized clinical features, hematologic parameters, serum vitamin B12 levels, and autoimmune associations in three siblings with GPS

Case number	Sex	Age at presentation	Current age	Clinical manifestation	Splenomegaly (ultrasound)	Hb (g/dL; ref: 11.5–15.5)	MCV (fL; ref: 80–100)	WBC (×10⁹/L; ref: 4–11)	Platelet (×10⁹/L; ref: 150–450)	PT (s; ref: 11–13.5)	aPTT (s; ref: 25–35)	Vitamin B12 (pg/mL; reference range 187–883)	Autoimmune association
Case 1	M	6 Yr.	11 Yr.	Gum bleeding, epistaxis, bruising after minor trauma	Absent	10.3	69	6	60	12.5	30	1469	Elevated serum IgG (2150 mg/dL)
Case 2	F	11 Yr.	16 Yr.	Mild bruising after minor trauma	Absent	11.2	72	6.3	93	12.8	31	1259	Yes (Type 1 Diabetes Mellitus & Celiac Disease)
Case 3	F	7 Yr.	12 Yr.	Bruising and prolonged epistaxis, irregular heavy menstrual bleeding at the age of 11 Yr.	Absent	12.7	75	7.7	125	12.6	30	1287	No

### Case 1

**Fig. 2:**
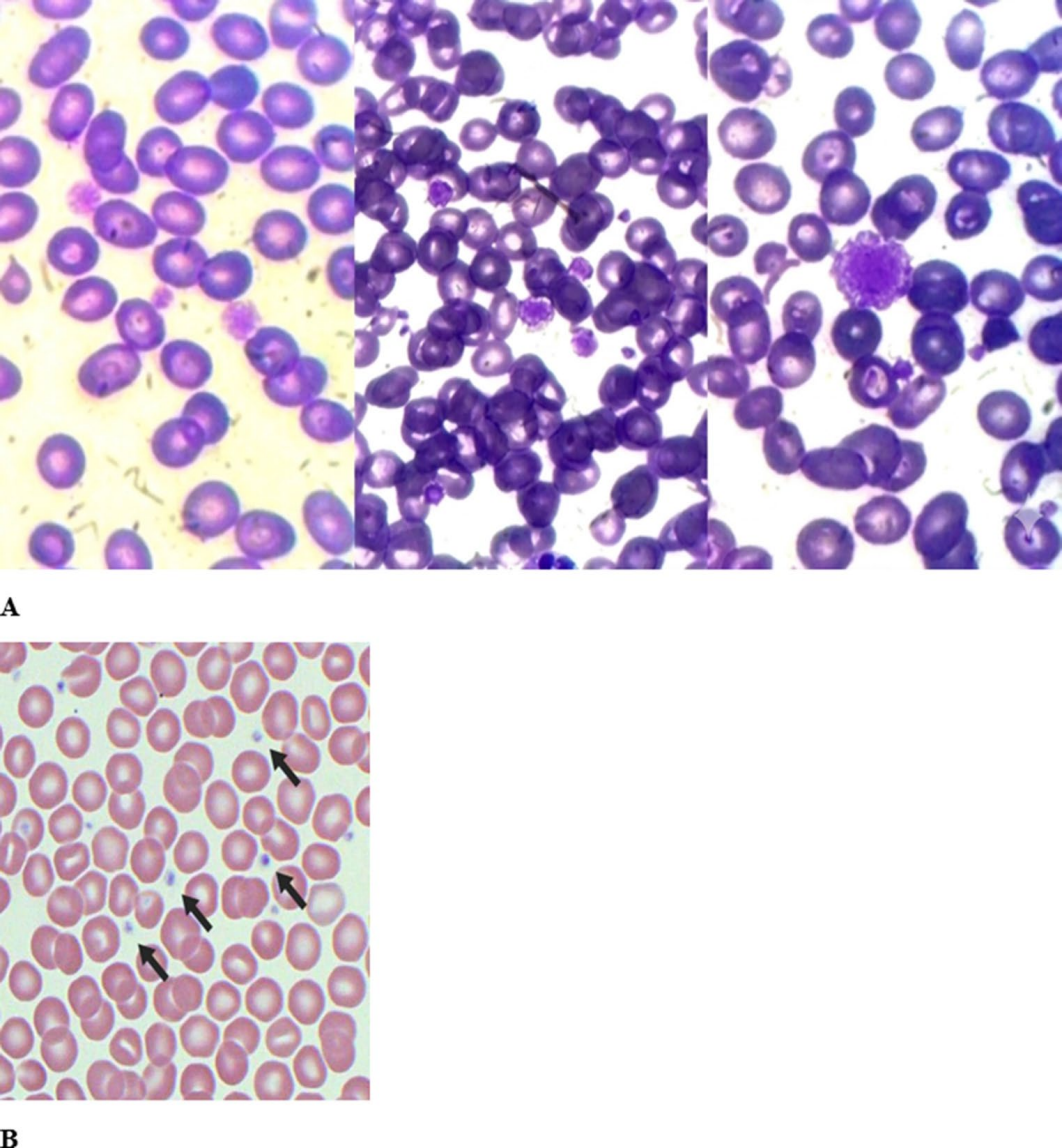
Peripheral blood smear comparison. **A** Peripheral blood smear from Case 1 demonstrating thrombocytopenia with enlarged, pale, hypogranular platelets consistent with gray platelet syndrome. Although the classic gray appearance is not well appreciated due to staining characteristics, the presence of large and hypogranular platelets remains evident. **B** Peripheral blood smear from a healthy control showing normal platelet morphology.

A 7-year-old boy presented with persistent gingival bleeding lasting three days following tooth extraction. Bleeding symptoms were first recognized in early childhood at approximately 2 years of age, with recurrent large bruises after minor trauma and intermittent epistaxis. On physical examination, he appeared pale and had cervical lymphadenopathy. However, no petechiae, dysmorphic features, or hepatosplenomegaly were observed. Laboratory evaluation revealed thrombocytopenia and iron-deficiency anemia, with prolonged bleeding time. Coagulation studies, including prothrombin time (PT) and activated partial thromboplastin time (aPTT), were within normal limits. Platelet function testing using light transmission aggregometry (including ristocetin, collagen, and ADP) was not performed due to limited availability of this assay at our institution. Peripheral blood smear demonstrated enlarged, pale, and hypogranular platelets, consistent with gray platelet syndrome, although the classic gray appearance may be less apparent depending on staining characteristics (Fig. 2). Peripheral blood smears for platelet morphology were prepared from samples collected in sodium citrate tubes. EDTA was not used, thereby excluding EDTA-dependent pseudo–gray platelet syndrome as a potential artifact. Bone marrow aspiration showed hypogranular megakaryocytes. Immunologic evaluation revealed elevated serum IgG levels for age (2150 mg/dL; reference range: approximately 700–1600 mg/dL), while IgM and IgA levels were within normal ranges. There was no clinical or laboratory evidence of acute infection or chronic liver disease.

Following the diagnosis of gray platelet syndrome, the patient was managed conservatively with bleeding precautions and iron supplementation. No regular platelet transfusions were required. No hemostatic agents, including tranexamic acid or desmopressin (DDAVP), were administered. During follow-up, he continued to experience mild mucocutaneous bleeding episodes but remained clinically stable without major hemorrhagic complications.

### Case 2

A 13-year-old female with a history of type 1 diabetes mellitus and biopsy-confirmed celiac disease since early childhood presented with mild bruising after minor trauma. Bleeding symptoms were first noted during early childhood but were mild and did not initially require medical evaluation. Thrombocytopenia was incidentally detected during hospitalization for an unrelated condition. Her clinical course has been relatively mild, with no history of significant mucocutaneous bleeding. Her clinical and laboratory findings are summarized in Table 1.

Following the diagnosis of gray platelet syndrome, she was managed conservatively with education regarding bleeding risk and avoidance of antiplatelet medications. No hemostatic medications, including tranexamic acid or desmopressin (DDAVP), were required. Her diabetes and celiac disease were managed according to standard treatment protocols. During follow-up, she remained clinically stable with no progression of bleeding symptoms.

### Case 3

An 11-year-old female presented with easy bruising and recurrent epistaxis, with symptoms first recognized at approximately 3 years of age. Over time, her bleeding tendency progressed, and she later developed irregular and heavy menstrual bleeding beginning at menarche, which required multiple hospital admissions and blood transfusions. Hematologic findings were comparable to those observed in the other affected siblings (Table 1).

Following diagnosis, management included supportive care with red blood cell transfusions during severe bleeding episodes and counseling regarding bleeding precautions. No platelet transfusions were administered. Hemostatic agents such as tranexamic acid or desmopressin (DDAVP) were not used. During follow-up, her condition remained clinically significant due to persistent menorrhagia, requiring close monitoring and multidisciplinary care.

Based on the strong family history, characteristic platelet morphology on peripheral blood smear (Fig. 2), and bone marrow findings, Gray Platelet Syndrome (GPS) was suspected. To confirm the diagnosis, whole-exome sequencing (WES) was performed using genomic DNA extracted from peripheral blood samples. Sequencing was carried out on an Illumina platform with a mean read depth of approximately 100× and > 95% coverage of targeted exonic regions. Bioinformatic analysis focused on genes known to be associated with inherited platelet disorders.

Whole-exome sequencing was performed in all three affected siblings. The analysis identified the same homozygous missense variant in the NBEAL2 gene: c.6515G > A (chr3:47,046,766 G > A), resulting in the amino acid substitution p.Arg2172His. The variant allele frequency (VAF) was consistent with homozygosity in each patient (approximately 95–100%). This variant is located within the highly conserved BEACH (Beige and Chediak–Higashi) domain of NBEAL2, a region known to be essential for vesicular trafficking and platelet α-granule biogenesis.

According to the ClinVar database, this variant is currently classified as a Variant of Uncertain Significance (VUS). However, variants affecting the same codon have previously been associated with Gray Platelet Syndrome. In particular, the nearby substitution NBEAL2 p.Arg2172Cys (c.6514 C > T) is classified as likely pathogenic in ClinVar. In addition, this variant has been previously reported in patients with Gray Platelet Syndrome in the cohort described by Sims et al. [9]. In silico prediction tools, including SIFT and PolyPhen-2, further support a deleterious effect on protein function. In the context of the characteristic clinical phenotype and segregation within this consanguineous family, these findings support the pathogenic relevance of this variant.

## Discussion

Gray platelet syndrome (GPS) is a rare inherited platelet disorder caused by pathogenic variants in NBEAL2, resulting in defective α-granule biogenesis and characteristic platelet morphological abnormalities [1]. Although historically regarded as a primary bleeding disorder, accumulating evidence supports the concept that GPS is a multisystem condition with prominent immune and inflammatory involvement [2, 9, 11]. In this familial case series, we describe three siblings with genetically confirmed GPS who exhibited not only classical hematologic features but also immune abnormalities, including autoimmune disease, polyclonal hypergammaglobulinemia, and persistent hypercobalaminemia, thereby expanding the recognized clinical phenotype of GPS.

Immune dysregulation has emerged as a central feature of GPS. Large cohort studies and systematic reviews have demonstrated increased frequencies of autoimmune and inflammatory conditions, including autoimmune cytopenias, inflammatory bowel disease, chronic inflammatory states, and immune mediated organ involvement in affected individuals [1, 2]. NBEAL2 is expressed across multiple hematopoietic and immune cell lineages, and experimental models have shown impaired granule formation in neutrophils, abnormal immune cell trafficking, and dysregulated cytokine signaling in the setting of NBEAL2 deficiency [3]. In our series, one patient demonstrated polyclonal elevation of serum IgG with normal IgM and IgA levels, in the absence of infection or liver disease, supporting chronic immune activation rather than a secondary reactive process. Hypergammaglobulinemia has previously been reported in GPS and is thought to reflect sustained immune stimulation related to defective intracellular trafficking and abnormal cytokine release [2, 4].

The most novel finding in our report is the coexistence of type 1 diabetes mellitus and biopsy confirmed celiac disease in a patient with genetically confirmed GPS. While multiple autoimmune conditions have been described in association with NBEAL2 deficiency, including endocrine (Hashimoto’s thyroiditis), musculoskeletal (rheumatoid arthritis), integumentary (alopecia, discoid lupus erythematosus, and vitiligo), and immune dysregulation disorders such as atypical autoimmune lymphoproliferative syndrome [2, 5, 6, 9], to our knowledge this is the first report documenting the concurrence of both type 1 diabetes mellitus and celiac disease in a single patient with GPS. These findings further expand the autoimmune phenotype associated with GPS and support the growing recognition of this disorder as a systemic condition involving immune dysregulation.

Recent reports have also described cases of Gray Platelet Syndrome originating from the Middle East. Notably, a recent case report from Palestine described a 14‑year‑old patient with GPS presenting with hepatomegaly and immune dysregulation [12]. Although the global prevalence of GPS remains extremely low, the occurrence of multiple reports from geographically related populations may raise the possibility of regional clustering or the contribution of consanguinity, which is relatively common in some populations and increases the likelihood of autosomal recessive disorders. As illustrated in the pedigree (Fig. 1), the disorder segregated within a consanguineous family, supporting autosomal recessive inheritance. Our report of three affected siblings further supports this possibility and highlights the importance of considering inherited platelet disorders in similar clinical contexts.

A consistent finding in our series was persistently elevated serum vitamin B12 levels in all affected siblings. Hypercobalaminemia is classically associated with myeloproliferative disorders, chronic inflammation, and immune-mediated conditions and is thought to result from increased circulating levels of vitamin B12–binding proteins, particularly haptocorrin, released by activated granulocytes and monocytes [7, 8, 10]. In GPS, NBEAL2 deficiency has been shown to affect granule formation not only in platelets but also in neutrophils and other myeloid cells, leading to abnormal immune activation and cytokine release [3, 6]. We hypothesize that elevated vitamin B12 in GPS may reflect chronic immune stimulation or altered myeloid cell function rather than true cobalamin excess. Elevated serum vitamin B12 levels have previously been described in patients with GPS, including in the cohorts reported by Gunay-Aygun et al. and Sims et al., and are thought to reflect chronic immune activation and increased release of cobalamin-binding proteins from activated myeloid cells [1, 9]. In our series, the consistent elevation of vitamin B12 across all affected siblings reinforces its potential role as a biomarker of immune dysregulation in GPS.

Although acquired forms of gray platelet syndrome have been reported in association with myeloproliferative neoplasms, particularly primary myelofibrosis, these cases typically occur in adulthood and lack a familial pattern [13]. In contrast, our patients presented with bleeding manifestations beginning in early childhood and had a clear family history involving multiple affected siblings from a consanguineous family, strongly supporting an inherited disorder. Whole-exome sequencing identified the same homozygous NBEAL2 variant in all three siblings using genomic DNA extracted from peripheral blood, which is consistent with a germline mutation rather than an acquired somatic alteration. Therefore, the clinical and genetic findings in our cases are most consistent with congenital Gray Platelet Syndrome.

## Conclusion

This familial case series expands the clinical spectrum of gray platelet syndrome and supports its classification as a multisystem disorder. It highlights the association of GPS with autoimmune diseases such as type 1 diabetes mellitus and celiac disease, further supporting the concept of immune dysregulation in this condition.

## Data Availability

No datasets were generated or analysed during the current study.

## Abbreviations

GPS: Gray platelet syndrome
PT: Prothrombin time
aPTT: Activated partial thromboplastin time

## Declarations

### Guarantor

Dr. Hussam Alzeerelhouseini.
